# Green tea and cocoa enhance cognition in *Lymnaea*

**DOI:** 10.1080/19420889.2018.1434390

**Published:** 2018-02-15

**Authors:** Erin Swinton, Emily de Freitas, Cayley Swinton, Tamila Shymansky, Emily Hiles, Jack Zhang, Cailin Rothwell, Ken Lukowiak

**Affiliations:** Hotchkiss Brain Institute, Cumming School of Medicine, University of Calgary, Calgary, Alberta, Canada

**Keywords:** *Lymnaea*, (-)-epicatechin, long-term memory, green tea, cocoa

## Abstract

A flavonoid, (-)-epicatechi (Epi), enhances long-term memory (LTM) formation in *Lymnaea* and reverses memory obstruction caused by stress. Many foods contain substantial amounts of Epi, (e.g. green tea and cocoa). In humans eating such foods may directly or indirectly enhance cognition. We directly test whether operant conditioning training *Lymnaea* in these natural foods result in the same effects as training snails in pure Epi. We found that exposure to products containing high concentrations of Epi (e.g. green tea and cocoa) during training enhanced memory formation and could even reverse a learning and memory deficit brought about by stress. Epi can be photo-inactivated by exposure to ultraviolet light. We found that following photo-inactivation of Epi, memory enhancement did not occur. Photo-inactivation of foods containing Epi (e,g. green tea) blocked their ability to enhance LTM. Our data are thus consistent with the hypothesis that dietary sources of Epi can have positive benefits on cognitive ability and be able to reverse memory aversive states.

## Introduction

The ability to form memory is ‘what makes me, me and you, you’. All aspects of memory formation, retention and recall are dynamic processes susceptible to modification by environmental factors, such as stress and lifestyle choices. One key lifestyle choice that may impact memory formation and retention is diet. Flavonoids are a group of phytochemicals found in plants that have been associated with cognitive enhancement in a wide variety of species including invertebrates [1–4]. (-)-Epicatechin (Epi), a flavonol found in many foods (e.g. green tea, cocoa powder and certain types of apple peels) is one of the most extensively studied phytochemicals [5,6] and is the focus of this present study.

The positive cognitive effects of flavonoids in mammals have been attributed to the protection of neural functioning, stimulation of neuronal regeneration, and increased blood flow to the brain [1,7,8]. Epi has been shown to possibly protect against neuronal death due to oxidative stress [9,10]. Thus, Epi is thought to enhance cognitive ability or prevent its decline. However, the specific cellular mechanisms directly linking Epi intake with enhanced cognition remains under investigation. Epidemiological studies suggest that Epi intake from food products such as cocoa correlate with a lower incidence of cardiovascular disease as well as decreased cognitive impairment seen in aged individuals [11–13]. Importantly much research has focused on flavonol consumption as a method of mitigating the devastating consequences of cognitive loss due to ageing [2,14].

Learning and memory are distinct but related cognitive processes. We define learning as a change in behaviour due to experience; and memory as the ability to recall this change and apply it in future circumstances [15]. We have been studying how memory in *Lymnaea* is modified by stress, age and bio-active products [16]. Long-term memory (LTM), which in *Lymnaea* persists for at least 24 h after training is dependent on altered gene activity and new protein synthesis [17]. Thus, changes in the ease to form LTM or to extend its duration brought about for example, by training in Epi, are thought to be caused by changes in altered gene activity or new protein synthesis [4].

*Lymnaea* can be operantly conditioned to reduce aerial respiratory behaviour [15,18]. Importantly, a single neuron in the central pattern generator (CPG) circuit that drives this behaviour has been shown to be a necessary site for LTM formation, memory extinction, reconsolidation of memory and forgetting [19–22]. These attributes make this model system ideal for examining how environmental stimuli alter memory formation [16,23]. These characteristics also allow us to observe the memory-enhancing effects of Epi [24]. In *Lymnaea* Epi purchased from Sigma (defined here as pure Epi) has been shown to enhance LTM formation following operant conditioning of aerial respiratory behaviour [3]. In addition, pure Epi has also been shown to produce similar memory-enhancing effects in *Lymnaea* when snails were exposed to it during the memory consolidation period [25]. Finally, Epi exposure immediately following a combination of stressors that obstruct all learning and memory processes reverses this occlusion and further enhances LTM formation [4]. However, these results have only been observed when the snails were exposed to pure Epi. Therefore, these results may not be representative of how Epi present in food influences memory formation and retention.

For this reason, we explore whether exposure to food products containing Epi content (e.g. green tea, cocoa powder and Red Delicious apple pee;l [5,26] during training in concentrations comparable to human consumption levels (approximately 1g/day) elicits similar effects that we have previously seen with pure Epi. To aid our investigation into whether food substances containing Epi have similar ability as ‘pure’ Epi in enhancing memory we make use of a finding that UVB light inactivates Epi [28]. Thus, we can test whether exposure to UVB light alters the ‘pure’ Epi effect as well as the effect on cognition brought about by food substances containing Epi. Here we investigated whether exposure: 1) to Epi containing products enhances LTM formation; 2) reverses the aversive stressors effect on learning and memory; and 3) to UV light blocks the pure Epi and high Epi content products enhancing effects on memory in *Lymnaea*.

## Results

### Aerial respiratory behaviour

In previous studies, it has been demonstrated that total breathing time is not altered by pure Epi [3]. Here we first determined if the pond water (PW)-containing food substances altered homeostatic aerial respiratory behaviour. We compared total breathing time (TBT) of the snails in PW and PW-containing food substances. Thus, we monitored TBT in 3 separate cohorts of snails. Each snail was only observed in PW, then 24 h later food substance-PW, then 24 h later in PW again. We found that TBT was not significantly different between PW and the three PW-containing food substances. The Repeated Measures (RM) one-way ANOVA for Cocoa (F(_1.586, 14.27)_ = 1.010; p = 0.307), Green Tea (F_(1.332, 11.99_ = 0.7046; p = 0.4578) and Red Delicious apple peel (F_(1.785, 16.07_) = 1.250; p = 0.3087) all show that the Epi-containing products at the concentrations used here did not significantly alter homeostatic breathing behaviour. Thus, that the concentration of Epi-containing food products used here did not alter their normal homeostatic response to hypoxic conditions.

### Memory enhancement of Epi product exposure

**Figure 1.**
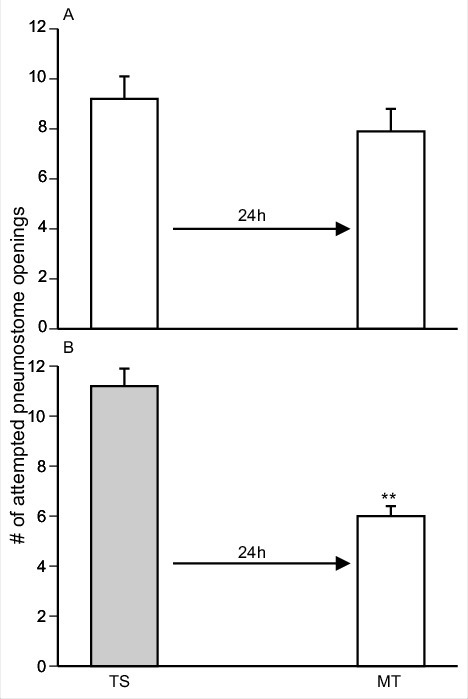
Training snails in PW and Epi with the single 0.5 h training procedure. A) A cohort (n = 11) of naïve snails was trained in PW (TS) and LTM (MT) was tested 24 h later. In B, another cohort of naïve snails (n = 17) was trained in a similar manner as in A, only in Epi-containing PW. LTM was not present in A but was in B. Thus, training in Epi-PW caused enhanced LTM formation. ** Significant difference between TS and MT (*P* < 0.01).

**Figure 2.**
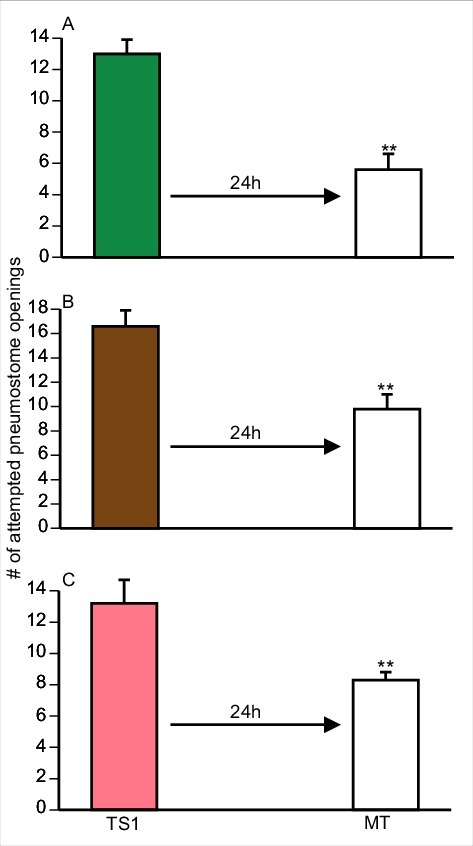
Training in green tea PW, cocoa PW and apple peel PW, all substances with a high Epi content, enhance LTM. Snails received a single 0.5 h training session (TS) in either green tea PW (green bar), cocoa PW (brown bar), or apple peel PW (pink bar) and were tested for LTM 24 h later. Each cohort trained in an Epi containing product in TS formed 24 h LTM defined by significantly fewer attempted pneumostome openings in MT compared to TS. ** Significant difference between TS and MT (P<0.01).

Snails exposed to pure Epi during one 0.5 h training session form LTM; while if trained in pond-water only do not [3]. We repeated these experiments with Epi pond water and pond water alone (Fig. 1A] and B]). We then examined whether epi-containing foods in pond water (i.e. green tea, cocoa, and apple peels; see methods) also cause the enhancement of LTM formation in snails. To do this we employed 3 separate cohorts of naïve snails: 1) a cohort trained in green-tea; 2) a cohort trained in cocoa; and 3) a cohort trained in apple peel. These data are presented in Fig. 2 A-C] respectively.

Here we define enhanced memory formation as the demonstration that LTM in the memory test session (MT) is present 24 h after training with a single 0.5 h training session (TS). As can be seen in Fig. 1] training in PW does not result in LTM while training in pure Epi-PW does.

We performed a 2-Way ANOVA followed by a Tukey's *post-hoc* test on the data presented in Figs. 1 and 2. These analyses showed that:

1) There was an interaction (F_(4,154)_ = 3.121; p = 0.0168) between the variables (i.e. the sessions ((TS and MT) and treatment (epi, PW, green tea, cocoa and apple-peel). When we performed a Tukey's *post-hoc* test we found the number of attempted pneumostome openings in the TS of the ‘cocoa-trained snails’ was significantly greater than the number of attempted openings in the TS of the pond water control and the Epi pond water control. Thus, the snails in cocoa pond water group received significantly more pokes in the TS than in the pond water and epi pond water cohorts. However, there were no difference in the TS between the cocoa, green tea and apple-peel cohorts in the TS.
2) The comparison of the five cohorts showed there was a significant difference between treatment of the groups (F_(4,154)_ = 5.389; p = 0.0004) as was there a significant difference when we compared the TS with the MT across the groups (F_(4,154)_ = 53.45; *p* < 0.0001).
3) Performing a Tukey's *post-hoc* test on these data we found that in all cohorts, with the exception of the pond water control, the MT in each cohort was significantly less than the TS. Thus, in all the cohorts, epi or epi-containing foods resulted in enhanced LTM formation. In addition, the MTs of the epi-pond water, green-tea, apple-peel and cocoa cohorts were not different. The PW-control MT was significantly different than the MTs in all other groups. We conclude that Epi or the natural food substances used here that contain epi cause enhancement of LTM formation in *Lymnaea*.

### Memory deficit reversal produced by Epi product exposure

It has previously been show that snails maintained in a low Ca^++^ environment (20 mg/l) for 1 week and then crowded (20snails/100 ml) immediately prior to operant conditioning training are unable to learn and form LTM [4,27]. However, if snails are similarly stressed and then trained (two 0.5 h training session separated by an hour) in Epi-low Ca^++^ PW, snails regain their ability to learn and form LTM [4]. Since exposure to green tea PW, cocoa PW and apple peel PW in the single 0.5 h TS caused enhancement of LTM formation (Fig. 2]); we asked whether training snails in PW containing the food substances with a high Epi content (Green tea, cocoa, apple-peel) is also able to reverse memory occlusion due to the combined effect of the low Ca^++^ and crowding stressors.

**Figure 3.**
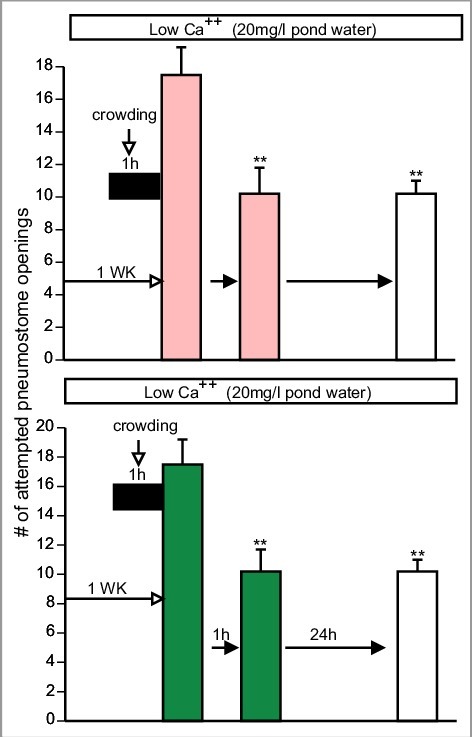
Food substances with high Epi content (apple peel PW and green tea PW) rescues obstructed memory formation. In sails, exposure to low Ca^++^ conditions (20 mg/L) for 7d and then crowded (20 snails/100 ml) immediately before training all memory processes are obstructed. Training snails in apple peel PW (top) or green tea PW (Bottom) reverses the memory deficit. In apple-peel low Ca^++^ PW (n = 10 F_(1.654, 9.924) = 8.213_ p = 0.0098) and green tea low Ca^++^ PW (n = 10 (F_(1.783, 16.05) = 15.67_ p = 0.0002) ITM and LTM are observed. That is, in both cohorts TS2 is significantly less than TS1 and MT is significantly less than TS1 and not significantly greater than TS2 (Tukey's *post hoc* test). ** Significant difference between TS1 and TS2 and MT (*P* < 0.01).

**Figure 4.**
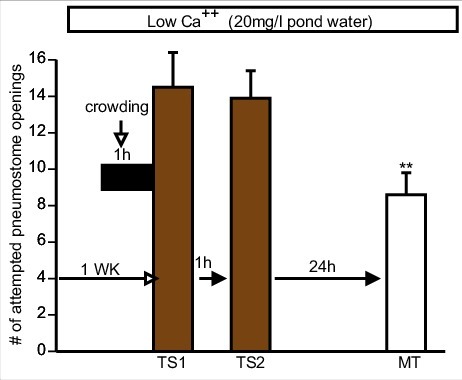
Cocoa containing PW rescues obstructed LTM formation. As in Fig. 3 snails (n = 11) were exposed to low Ca^++^ conditions (20 mg/L) for 7d and crowded (20 snails/100 ml) but were now trained in cocoa low Ca^++^ PW. An ANOVA F_(1.329, 13.29) = 7.828_ p = 0.0103 shows that LTM was formed as the Tukey's *post hoc* test showed that MT was significantly less than TS1 and not significantly greater than TS2. However, the Tukey's *post hoc* test showed that TS1 and TS2 were not significantly different. We think this occurred due to an increase in feeding behaviour (rasping), therefore the snails were too “over-stimulated” to show learning and ITM. ** Significant difference between TS1 and MT (*P* < 0.01).

For each Epi-containing product PW (i.e. green-tea (n = 10), apple peel (n = 10) and cocoa (n = 11)) Figs. 3 and 4), three new naive cohorts of snails were kept in low Ca^++^ PW for 1 week and then crowded (20snails/100 ml) for 1 h immediately before being trained in low Ca^++^ PW containing the Epi products. Each cohort of snails was trained in their respective product (two 0.5 h training session separated by an hour) and LTM was tested 24 h later in low Ca^++^ PW (i.e. the epi-containing product was not present in the MT). A 2-Way ANOVA followed by a Tukey's *post-hoc* test was performed on these data. These analyses showed that:

1) There was not an interaction (F_(4,84)_ = 1.617; p = 0.1776) between the variables (i.e. the sessions ((TS1, TS2 and MT) and treatment (green tea, cocoa and apple-peel).
2) The overall comparison of the three cohorts showed there was no difference the number of (attempted pneumostome openings in the two training sessions and the memory-test sessions ((MT) respectively between the cohorts (F_(2,84)_ = 0.444; p = 0.9566).
3) Finally, a comparison in the three (cohorts showed there were significant differences between the TS1 and MT in each of the three groups; meaning that LTM formation occurred in each cohort (F_(2,84)_ = 14.26; *p* < 0.0001).

Thus, the food substances with high Epi content were able to rescue the learning and memory processes occluded by the combined low Ca^++^ and crowding stressors.

However, the Tukey's *post-hoc* analysis of the cocoa data in this experiment (Fig. 4]) showed that the number of attempted pneumostome openings in TS1 and TS2 were not significantly different from each other; whereas in both the green-tea and apple-peel experiments (Fig. 3]) the number of attempted openings in TS2 was significantly less than in TS1. Since in the cocoa experiment TS2 was not significantly different than TS1, learning and intermediate-term memory ITM did not appear to have been made. However, as stated above when we tested 24 h later if memory had been formed in PW it was present. In performing this experiment, we observed increased rasping behaviour in cocoa PW during the two training sessions. We hypothesized that this increase in rasping behaviour, which is indicative of an increased feeding response, could have occluded the ITM. That is, in the TS2 session the increased feeding response elicited by cocoa may have obstructed ITM. Therefore, we investigated how each of the Epi containing products alter rasping behaviour in *Lymnaea*.

### Rasping behaviour in the high Epi content substance PW

**Figure 5.**
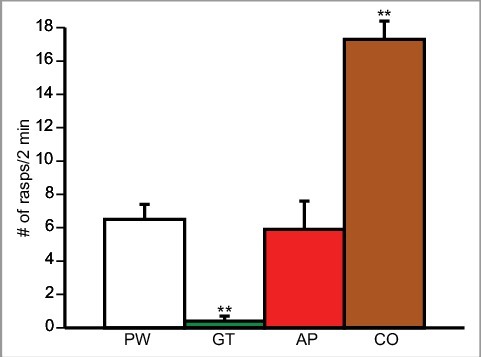
Rasping behaviour and food substances high in Epi content. Snails (n = 10) were exposed to the Epi containing food substances and their rasping behaviour was observed to each food substance. Plotted are the number of rasps/2 minute period. An ANOVA F_(2.729, 24.56) = 27.76_ p< 0.0001). The Tukey's *post hoc* test showed that there was a significant difference between the number of rasps in PW compared to apple peel PW and between PW and cocoa PW. While exposed to cocoa water, snails showed a significant increase in rasping behaviour compared to when exposed to PW. The number of rasps/minute was significantly lower in apple peel PW compared to PW and no change in rasping behaviour was observed between green tea PW and normal PW. ** Significant difference between PW and apple peel (AP) and between AP and Cocoa (CO) (P<0.01).

A group of naïve snails (n = 10; Fig. 5]) were exposed to normal PW and then in random order to each Epi-containing product in a petri dish. Thus, 4 petri-dishes were used. After a 10 min acclimation period, the number of rasps/two minutes was counted. We observed a significant increase in the number of rasps between PW and cocoa PW and a significant decrease in rasping behaviour in green tea PW. In contrast, the number of rasps was not different between PW and apple-peel PW. This observation is consistent with the hypothesis that snails being trained in low Ca^++^ cocoa PW (Fig. 4]) have an increased feeding response that occludes learning and ITM.

### Photo inactivation of epi containing products

In the food products we tested here there are other bioactive compounds which possibly could affect LTM formation. To attempt to control for this we choose a strategy of photo-inactivating Epi in these food substances and then observe if the food substances still enhanced LTM formation. A recent published study [28] successfully inactivated Epi in tea by exposing it to beta-UV radiation. This is equivalent to exposure to the sun for six hours.

**Figure 6.**
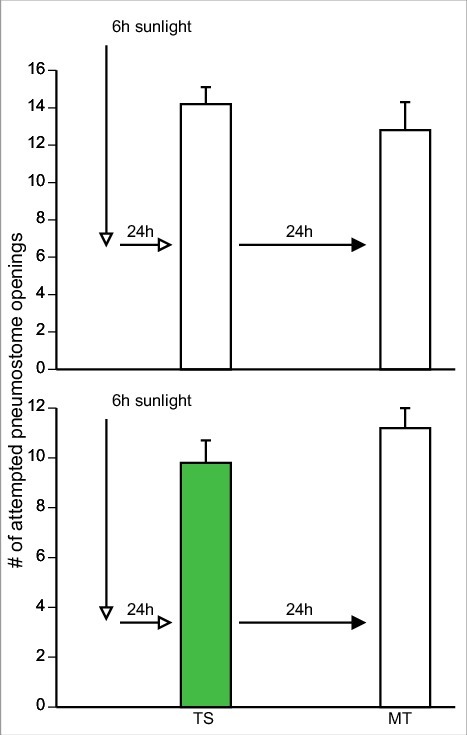
The effect of UV exposure on Epi PW and green tea PW. Snails were trained in photo-inactivated PW containing Epi (n = 18, top) and green tea (n = 19, bottom, green bar), with a single 0.5 h TS and memory tested 24 h later (MT). There was no significant difference between the number of pneumostome openings in TSI compared to MT in the photo-inactivated Epi (t = 0.8935; p = 0.3841) or photo-inactivated green tea PW (t = 1.388, p = 0.1822).

**Figure 7.**
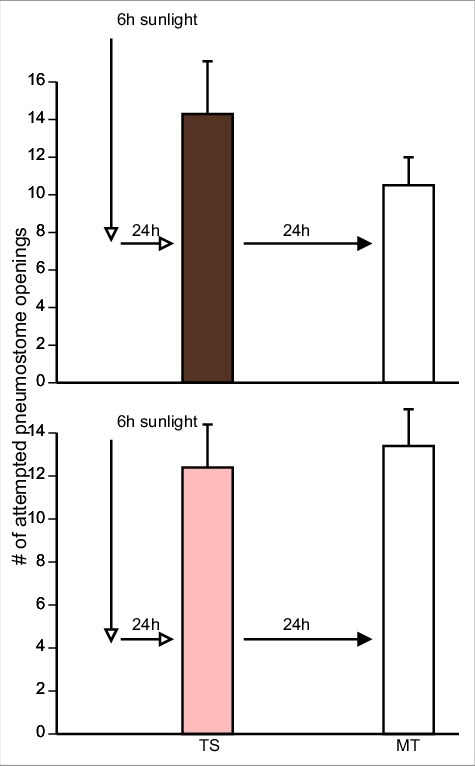
The effect of UV exposure on cocoa PW (n = 10, top, brown bar; t = 1.495, p = 0.1692) and apple peel PW (n = 11, bottom, pink bar; t = 0.5388, p = 0.6018). As in Fig. 6. Snails trained in photo-exposed food substance PW no longer exhibited enhanced memory forming abilities.

**Figure 8.**
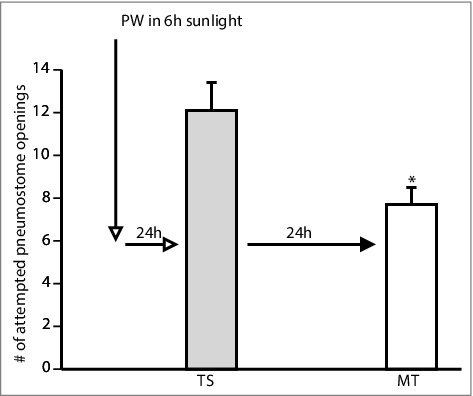
Photo-exposed PW and Epi added 24 h later enhances LTM formation. PW was exposed to UVB radiation and then 24 h later, pure Epi was added. A naive cohort of snails (n = 9) was trained in this Epi PW. LTM formation was observed.(TS vs. MT = t = 2.443, P = 0.0404). * (P<0.05).

We therefore exposed pure Epi PW(n = 18) and the three substances (green tea PW (n = 19), cocoa PW (n = 10), and apple peel PW, n = 11) used above to the photo inactivation procedure. Following this procedure, we trained the four separate cohorts of naive snails in the sun-exposed PWs. As an additional control, we subjected PW to the photo inactivation procedure and then added pure Epi to it and trained a fifth cohort of naive snails (n = 9). Thus, 5 separate cohorts of naïve snails were trained using the single 0.5 h training procedure and memory was tested 24 h later (Figs. 6,7,8).

A 2-Way ANOVA followed by a Tukey's *post-hoc* test was performed on these data. These analyses showed that:

1) There was not an interaction (F_(4,124)_ = 0.1167; p = 0.9764) between the variables (i.e. the sessions ((TS and MT) and treatment (sun exposed green tea, cocoa, apple-peel and PW then added epi).
2) The overall comparison of the 5 cohorts showed there was no difference in the number of (attempted pneumostome openings in TS and MT respectively between the cohorts (F_(4,124)_ = 0.1606; p = 0.9578).
3) A comparison in the 5 (cohorts showed overall there was no effect of trail on the number of attempted pneumostome openings (i.e. TS was not different from MT) (F_(,124) = 0.6802_; p = 0.6802). However, when we examined the result of the Tukey's *post-hoc* test we found that the MT was significantly less than TS in the cohort that had PW exposed to sunlight then epi added. Thus, in that cohort epi enhanced LTM formation.

These data allow us to conclude that the UV-exposure procedure used here abolishes the ability of Epi-PW and food substances-PW to enhance LTM formation. However, the UV-procedure performed on PW to which Epi is later added to it still causes memory enhancement.

Table 1] summarizes the effects on LTM formation of Pure Epi, food substances containing epi, and what the exposure to UV light does.

**Table 1.** Results of the 2-Way ANOVA on differences in treatment on memory formation.

Treatment	TS vs MT
PW	NSD
Epi in PW	**
Green tea PW	**
Cocoa PW	**
Apple Peel PW	**
Apple Peel on low Ca^++^ and Crowding	**
Green tea on low Ca^++^ and Crowding	**
Cocoa on low Ca^++^ and Crowding	**
Sun + PW then Epi	**
Sun + Epi	NSD
Sun + Green tea	NSD
Sun + Cocoa	NSD
Sun + PW then Epi	**

## Discussion

Our results support the hypothesis that the flavonol, (-)-epicatechin (Epi), found in abundance in green tea, cocoa powder and apple peel elicit Epi-modulated enhancement of memory in our *Lymnaea* model system. The results obtained with the high Epi-content food substances are comparable to the memory enhancement elicited by pure Epi in our previous experiments [3,25,32]. It has previously been shown that *Lymnaea* respond with changes in memory formation to many other bio-active compounds (e.g. H_2_S, cocaine, and methamphetamin;e [3,29–31]. Pure Epi was also shown to reverse memory occlusion elicited by memory “aversive” stressors [4,32]. Presently, it is unclear how epi alters neuronal activity that causes the enhancement of LTM formation, but preliminary data shows that it is an activity dependent process requiring tactile input of the pneumostome area.

Epi is found in abundance in some fruit and vegetable products [26], among them green tea, cocoa, and Red Delicious apple peels. This raises the question whether *Lymnaea* would show similar cognitive enhancement, as those brought about by pure Epi, if trained in PW made from these food products. Since the memory enhancing effects of many bioactive compounds on *Lymnaea* often mirror those seen in mammals [30,31], *Lymnaea* could successfully serve as a model to examine the effects of Epi containing products consumed by humans on cognitive ability. Here we hypothesized that exposing snails to high Epi containing food products such as green tea PW, cocoa powder PW and apple peel PW during training would lead to memory enhancement. We demonstrated this by: 1) naïve snails trained with a single 0.5 h training session in all three high Epi content food substances show enhanced LTM; 2) when snails were put into a “memory aversive” state by exposure to two stressors (i.e. low calcium PW and crowding) the food substances reversed the stressor-induced memory occlusion; 3) pure Epi and the food substances used here when exposed to UVB radiation no longer cause LTM enhancement. Together these results are all consistent with the hypothesis that dietary sources of Epi have positive effects on memory formation. It is important to remember that the concentration of ‘Pure’ Epi and the dilutions of the food substances used did not alter important homeostatic functions such as aerial respiration.

However, two of the food substances used here, green tea and cocoa, alter the number of rasps (i.e. feeding movements) made by the snails. Snails in Green-tea PW rasp significantly less than they do in PW. On the other hand, the same snails in Cocoa-PW rasp at a significantly higher rate (about 3 times more) than they do in pond water. We hypothesize that this increase in rasping behaviour may be the reason why when snails were trained using the two-session procedure (Fig. 4]) we did not observe a significant decrease in the number of attempted pneumostome openings between TS1 and TS2. The cocoa-PW obscured the memory at that point, even though it was present. However, it is important to note that when tested 24 h later in the low calcium pond water these snails showed LTM. It may be that the snails in cocoa-PW exhibit the ‘Necessity knows no law’ concept. It has previously been shown [55-57] that severely food-deprived snails apparently do not learn and show do exhibit conditioned taste aversion (CTA) memory. However, as soon as they are feed they exhibit CTA memory. It appears that LTM formed but was ‘overpowered’ or occluded by the effects of food deprivation. Remove the stress associated with food deprivation and memory becomes retrievable. The substance(s) in cocoa and green tea that alter the neuronal activity that drives rasping behaviour are not known but we know that these substances do not affect the molecular events in neurons necessary for LTM formation. Future studies will examine the mechanisms which underlie the negative effects produced by green-tea and the positive effects on rasping that cocoa PW has.

Ingestion of food substances with a high Epi and other flavonol content have all been cited to have significant health, benefits, including enhanced cognition, to humans [1,6,14]. The two most commonly cited explanations for their positive effects on cognition are their high anti-oxidant content and their positive angiogenic effect [33–35]. The production of free radicals in neurons results in oxidative damage at a cellular level and this impacts cognitive ability both humans and *Lymnaea* [36,37]. Epi possesses anti-oxidant properties, which is hypothesized to protect neurons from injury caused by oxidative stress [10]. Epi's ability to cause blood vessel formation was also thought to indirectly alter cognition by increasing blood flow to the brain [33,38]. However, we believe that Epi's effects on cognition in *Lymnaea* are not the result of Epi's anti-oxidant or angiogenic effects since the changes (i.e. ability to enhance LTM) occur within 30 min and since *Lymnaea* possess an open circulatory system.

In mammals Epi is able to cross the blood-brain barrier in a time-dependent, stereo-selective manner and is present in brain tissue in higher concentrations after ingestion [7]. Because Epi's effects in *Lymnaea* occur within a short period of time (30–40 min) and are only effective in enhancing LTM formation when present during training or immediately after training [3,25], it is thought that Epi has a direct effect on neurons necessary for LTM formation [4]. This in part suggests to us that in *Lymnaea* the memory enhancing effects of Epi exposure are unlikely to be due to its anti-oxidant properties. Firstly, exposing *Lymnaea* to Epi elicits an immediate effect on their memory forming capabilities. The combination of two stressors (low Ca^++^ and crowding) obstructs all memory-related processes including short-term and intermediate-term memory [27]. However, these memory processes are immediately rescued when snails were trained in pure Epi PW. These results were also shown here when snails were trained in green tea PW, cocoa PW and apple peel PW immediately after snails were exposed to “memory unfriendly” stressors. Moreover, here we observed that Epi, a photosensitive flavonol, does not elicit memory enhancement in *Lymnaea* upon being inactivated by UVB radiation. Previously, it was shown that the antioxidant properties of Epi do not significantly change upon being altered by sunlight exposure [28]. Thus, the memory enhancement seen with pure Epi and Epi-containing foods exposure observed here in *Lymnaea* is unlikely to be caused by Epi's antioxidant properties and is more likely due to a direct effect on signaling cascades necessary for memory formation in neurons necessary for LTM formation. However, this does not mean that it's antioxidant properties do not prevent oxidative damage and therefore prevent neuronal damage over the long-term.

We are uncertain what the exact concentration of Epi or even if there is Epi is in the food substance-PWs used in our experiments. We have used a bio-assay approach picking a dilution that alters neither normal homeostatic respiration or locomotion but which brings about a similar effect on enhancement of LTM formation that a concentration of 15mg^−/l^ does. We do not have the equipment or the expertise to measure epi in the various food substances PWs. As was pointed out (Bhagwat and Haytowitz 2015) [26] how food substances such as green tea is made varies greatly across cultures and individual preferences. Thus, it is difficult to compare flavonoid contents obtained from different sources. We attempted to follow the procedures outlined in that paper to obtain a content of epi that was ‘ball park’ to what we used in the ‘pure’ epi experiments. In the studies of epicatechin effects on cognitive ability [12,13,14] in humans, the authors prepare cocoa much as we did here, so we are fairly confident that for green tea and cocoa infusions there is epi present in the PW. We arrived at using 4-day apple peel water because we were attempting to try to get the snails to eat the apple-peels. After being in the aquarium for 4 days with apple peels we noticed that the apple peels were not eaten. So, in a pilot experiment we tested whether this pond water enhanced LTM formation. It did. Thus, we used this method. Whether longer or shorter periods would result in different effects are unknown. We also, based in part on the data with green-tea pond water and rasping that ingestion of epi is not the primary manner in which epi gets into *Lymnaea*. In our initial experiments on What we do know is that the three food-substance pond waters used here result in the same effects on LTM formation as the concentration of epi used here and previously [3,4].

Our findings that different food substances can alter memory enhancement led us to ponder if a difference in plants found in ponds could help to explain differences in cognitive ability between populations of snails. We have found that freshly collected snails from some ponds can be characterized as ‘smart’ while others have been characterized as ‘average’ [58,59]. We do not believe this to be the cause of ‘smart vs average’ for the following reasons: 1) The differences in cognitive ability between the different strains have been maintained in the laboratory for many years even though all strains are feed on supermarket purchased romaine lettuce; and 2) In experiments on ‘average’ juvenile snails; training these snails in Epi enhances LTM formation. However, when examined as adults they do not exhibit the ‘smart’ phenotype.

A possible mechanism explaining the positive cognitive effects of Epi exposure in mammals is increased perfusion of the brain via an increase in angiogenesis [1,14,33]. In addition, flavonoids have been shown to aid in the production of other signaling molecules such as nitric oxide (NO) and insulin. NO inhibits inflammation and improves vascular endothelial function, thereby increasing brain blood perfusion [8]. NO has been shown to alter neural function in *Lymnaea* [39,40] but whether an NO increase occurs with Epi exposure in *Lymnaea* is not known. Epi's positive effect on insulin signaling in mammals possibly also plays a role in increasing cognitive ability or slowing down cognitive decline seen in aged individuals [14]. In *Lymnaea*, insulin has been shown to play a key role in memory formation [41–43] but whether Epi has any effect on insulin production or its activity in *Lymnaea* has so far not been investigated. Although the exact molecular process behind how Epi plays a role in cognition is unknown, research has shown that flavonoids such as Epi can interact with a number of cellular signaling cascades, primarily with mitogen-activated protein cascade [3–5]. These cascades trigger gene expression and protein synthesis necessary for eliciting/maintaining the cellular neuronal changes underlying memory formation [5].

Here we were able to replicate the memory enhancing effects of pure Epi as well as pure Epi's ability to reverse a “memory unfriendly” state by using high content Epi foods. Our results are consistent with the hypothesis that it is Epi in the natural food substances (e.g. green tea PW) that is having the positive effect on memory. In addition to producing immediate memory enhancement, exposure to Epi containing foods was able to overcome a ‘memory aversive’ state, indicating that food rich in flavonoids may be capable of reversing or preventing states such as ageing where memory formation and retention can be compromised. Whether Epi will alter a memory deficit state seen in aged *Lymnaea* [37] needs to be determined and these experiments are planned in the future. The results presented show for the first-time beneficial effects of natural foods on memory formation in *Lymnaea* and thus encourage us to use memory formation in *Lymnaea* as a model system to study how natural foods may enhance memory formation or counteract memory-deficit states in humans.

## Materials and methods

### Animals

Adult *Lymnaea* (25 mm spine height) originating from the Dutch laboratory strain collected in the 1950s from the canals in a polder located near Utrecht were used. These snails (the W-strain) were maintained in artificial pond water (PW, 0.25 g Instant Ocean, Spectrum Brands, Madison, WI, USA) and supplemented with CaCO_3_ to ensure Calcium concentrations remained above 50 mg [44]. Snails were fed romaine lettuce *ad libidum* and maintained @ 20 ± 1°C on a light: dark cycle of 16h:8h.

### Drug exposure

Pure Epi obtained from Sigma-Aldrich (St Louis, MO, USA) was used at the same concentration in PW (15 mg l^−1^) as in previous studies [3,4,25,32]. This concentration of Epi does not alter respiratory and locomotor homeostatic behaviour of the snails and is considered equivalent to human consumption levels.

Significant concentrations of Epi can be found in many naturally occurring products consumed by humans including green tea, cocoa powder and Red Delicious apple peel [5,26]. We attempted to test whether the Epi concentration in these natural products have effects on memory forming abilities in *Lymnaea* similar to pure Epi by exposing the snails to water containing the food products mentioned above.

For the green tea experiments, we used ‘Sensations Green Tea’ by Compliments from the grocery store Sobeys (Mississauga, ON, Ca). We prepared the green tea by boiling 250 ml PW and then steeping the green tea in PW for 2 minutes. The tea bag was then removed and the green tea was left to cool to room temperature (∼20±1°C). Once the green tea had cooled to room temperature, a 1:4 green tea: PW mixture was used to train snails in, using our normal operant conditioning protocol.

Cocoa powder has a high Epi content along with other polyphenolic bioactive compounds [7]. In order to test cocoa's effect on memory formation, we boiled 250 ml of PW before mixing it with 1 g of Fry's alkalized cocoa powder (purchased at Sobeys). The mixture was then left to cool to room temperature (20±1°C) before we prepared a 1:4 cocoa: PW mixture to be used in our operant conditioning training procedure.

Apple peels from Red Delicious apples has a high Epi content [45]. In order to expose the snails to Epi derived from apple peel, we peeled 1 g of Red Delicious apple peel and placed it in 1000 ml of PW for four days prior to training. The 4 day period was based on data we obtained from pilot experiments. We arrived at this time because we attempted to get snails to eat the apple peel and put snails and apple peels together in a small aquarium. The snails did not eat the apple peels. However, we tried in the pilot experiment to see if the pond water containing the apple peels would have any effect on LTM formation in the snails. It did. That is why we used the 4-day procedure. They did not. This allows time for the Epi to leach into the water. This ‘apple-peel’ PW was then used in our operant conditioning procedure.

The dilution factor and amount of the food product was chosen in order to represent the probable human consumption of Epi without significantly altering homeostatic aerial respiratory behaviour of the snails.

### Rasping behaviour

Feeding behaviour or rasping in *Lymnaea* is a rhythmic motor behaviour in which repeated movements of the radulae scrape the surface of a substrate leading to the ingestion of food [46]. Here the animals are exposed to the three food products used in the operant conditioning experiments to determine how they affect rasping behaviour. Snails were placed into a petri dish with a 14 cm diameter with enough of normal PW, cocoa PW, green tea PW, or apple peel PW for the snails to be fully submerged. The snails were given a 10-minute acclimatization period and then each snail was monitored for two minutes and number of rasps counted. The average number of rasps per 2 minute was then calculated. Once all snails had been monitored, they were removed from petri dish and placed in their home aquaria. Snails were given a rest period of one hour between each substance; substances were presented in a random order.

### Aerial respiratory behaviour

*Lymnaea stagnalis* are bimodal breathers. In eumoxia they acquire oxygen directly across their skin (i.e. cutaneous respiration) and as hypoxia develops they perform aerial respiration exchanging atmospheric air with air in their lung via the pneumostome [18,47–50]. Aerial respiratory behaviour is an easily monitored and adaptable behaviour. Pure Epi at the concentration used here does not alter aerial respiratory behaviour [3]. However, it has not been previously investigated whether exposure to green tea PW, cocoa PW, or apple peel PW as prepared here impacts breathing behaviour. To investigate whether these products influence aerial respiratory behaviour in *Lymnaea*, the total breathing time (TBT) was measured under hypoxic conditions over a 30-min period in normal PW, then one of green tea PW, cocoa PW and apple peel PW separately and then normal PW pond again after product exposure.

Naive cohorts of snails were placed in 1l beaker filled with 500 ml of PW or green tea PW, cocoa PW and apple peel PW, which had been made hypoxic by 20 min of vigorous N_2_ bubbling. Bubbling was reduced and maintained at a low level while observing breathing behaviour to maintain the hypoxic environment without disrupting snail activity. Snails were removed from their home aquaria and placed into the beaker where they were allowed to acclimatize for 10min. After the acclimatization period, the snails were observed for 30 min while recording breathing behaviour. Each individual was firstly observed in PW, then in PW containing a food product and in PW after food product exposure.

### Operant conditioning protocol

The strain of *Lymnaea* used in these experiments (i.e. Dutch snails) require two 0.5 h training sessions separated by 1 h to form a 24 h LTM [15,51]. Memory has been operationally defined [18] as the number of attempted pneumostome openings in the memory test session (MT) being significantly less than the number of attempted openings in the first test session (TS1) and not greater than the number of attempted openings in the second test session (TS2). If only a single 0.5 h training session is given (TS) than the MT 24 h or more later must be significantly less than in TS for us to conclude that memory has formed.

To train snails we make 500 ml of PW or PW containing a food substance hypoxic as described above. Snails are placed in the hypoxic environment for a 10 min acclimation period, followed by a 0.5 h training session. As the snails come to the surface and begin to open their pneumostome a tactile stimulus (a sharpened wooden stick) is applied to the pneumostome (i.e. a poke). The stimulus is sufficiently strong to cause closure of the pneumostome but gentle enough so as not to cause a full-body withdrawal response [18]. The number of attempted pneumostome openings is recorded for each individual snail over the 0.5 h period. In order to determine whether a 24 h LTM was formed following the single 0.5 h TS, LTM was tested for similarly 24 h later.

Previously we showed that pure Epi enhanced LTM formation in the Dutch snails as a single 0.5 h training session is now sufficient to result in LTM [3]. If the food substances containing a high content of Epi have the same effect on memory formation as pure Epi, we expect that a single 0.5 h TS will result in LTM when tested 24 h later.

### Low calcium and crowding stressor

As mentioned above, a stressor in the snail's external environment impacts the snail's ability to form memory. Some stressors for example, exposure to 30°C PW or predator scent (CE) enhance LTM formation [16,51–53]. However, this is not the case for all stressors. Some stressors obstruct LTM formation. For example, low Ca^++^ PW. In low Ca^++^ PW (Ca^++^ < 20 mg/l) LTM formation is blocked even though the snail still shows learning [54]. In a similar manner when snails are crowded together (20 snails/100 ml) LTM formation is obstructed but learning still occurs [55]. If snails experience both these stressors together both learning and memory formation are blocked [4,27].

Training snails in pure Epi in low Ca^++^ PW after experiencing crowding reverses the learning and memory forming deficits [4]. That is, learning and LTM occur. Whether the food substances containing Epi will produce a similar result will be determined here. We examined this by exposing naïve snails to low Ca^++^ PW (20 mg/l) for one week and then crowd them for 1h. They were then trained in low Ca^++^ PW containing the food substance. Memory was then tested 24 h later in low Ca^++^ PW.

### Epi Photo inactivation

Previous studies have shown that exposing pure Epi to UVB radiation similar to the intensity of the sun changes the conformation of the molecule by breaking the cyclic ether through a radical mechanism thus inactivating Epi [28]. We exposed Epi PW and the food substances PW to natural sunlight for 6 h in transparent glass containers before returning them to the lab. The snails were trained for 0.5 h in the photo-inactivated pure and Epi products using our standard single 0.5 h training procedure protocol. LTM was then tested 24 h later to determine whether the snails were able to form enhanced memory with photo inactivated Epi. This will help us to determine if Epi is the enhancing agent in the food substances as these substances contain many other bioactive compounds that could potentially impact the neural processes of memory formation or mechanistic action of Epi.

### Statistical analysis

All data were analyzed in GraphPad Prism, which allowed for between-subject comparisons for the effects of the treatment. A repeated measures one-way ANOVA's with multiple comparison analyses were employed to compared total breathing time among pre-exposure conditions, the treatment conditions and post-exposure conditions. This test was also used to determine significant differences in rasping behaviour in PW and other Epi containing products. 2-way ANOVA followed by a Tukey's *post-hoc* test was used to determine if there were differences in treatment between cohorts and if memory formed in Epi and Epi containing food substances-PW. Significance define as *p* < 0.05.
